# Supplementary material for: Reliability and validity of a low-cost, wireless sensor and smartphone app for measuring force during isometric and dynamic resistance exercises
Source: PLoS One. 2024 Mar 21;19(3):e0298859. doi: 10.1371/journal.pone.0298859 (PMC10956869; doi:10.1371/journal.pone.0298859)
Supplement: Attachment — Submitted filename: Response to Reviewers.docx [file pone.0298859.s002.docx]

**Responses to Reviewer #1**

Please note that:

**[R1] = comments of Reviewer #1.**

[A] = answers of the authors.

Reviewer #1: General:
The manuscript is extremely well written, with zero glaring English or formatting issues.
There are no major issues with this manuscript as each section does a great job and is of high quality. For this is commend the authors, and can only come up with minor revisions!
However, I must also include that there are several other modern strain-gauges (or similar) that have recently been examined for validity and reliability. Therefore, this study is not particularly special. Although I do recognize the unique way that the researchers examined dynamic force output through the use of elastic bands!

**Abstract:**

**[R1] = Not a big issue, but not sure why the abstract is broken into separate paragraphs.
Line 35: I’m not sure if bias can actually be zero. Might this be better expressed as “<0.01” or similar?**

[A] = Thank you for your comment. We have combined the paragraphs of the abstract without spaces.

Regarding line 35, we have indeed found other studies with a Bias of 0.00, and they represent it in a similar manner, including, as in our case, the confidence interval. We are attaching some references that have also found and reported a Bias of 0.00.

Adamakis, M. (2021). Criterion validity of iOS and Android applications to measure steps and distance in adults. *Technologies*, *9*(3), 55.

**[R1] = The abstract should be clearer regarding the isometric and dynamic ‘vertical row’. Would this be more accurate to say ‘upright row’?**

[A] = Thank you for the observation. We have replaced "vertical row" with "upright row in the abstract and throughout the document.

**Methods:**
**[R1]** = **The sample size of 13 is relatively small, especially for a fairly simplistic study. It is not a huge flaw, however, if the paper is not accepted, I would recommend the authors increase their sample to ~20 (unless they can present a compelling argument for why 13 is sufficient.**

[A] = Thank you for your input. We are aware that the sample size was small, and we could add this as a limitation in the manuscript. However, we have calculated the statistical power of our results for correlation analysis with this sample. Using the lowest correlation seen in the results (0.94, Fpeak at 120BPM) for the sample size used, the resultant statistical power is 0.98. This is higher than the usual 0.8 recommended power.

The power calculations were performed using the R open-source software through the 'pwr' library, as indicated in the following code snippet. library(pwr): pwr.r.test Power calculations for correlation test

pwr.r.test(n = 13, r = 0.94, sig.level = 0.001, power = NULL)

n = 13

r = 0.94

sig.level = 0.001

***power = 0.98372***

**[R1] = Line 117: I am not sure what the authors mean by “…resisted by a slack at…”
The use of elastic bands is very cool!**

[A] = We appreciate your suggestion, as it was not a clear term. Whe have therefore substituted the term “slack” for “non-elastic strap”, in order to improve clarity. It has also been changed in figure 1.

**[R1] = I really like the choices of statistical analysis! I am almost certain the figures were not made in JASP. Thus the authors should state how the figures were made.**

[A] = Thank you for acknowledging and appreciating this effort. The graphs were created using the GraphPad Prism software. We have included an statement for this fact at the end of the statistical analysis section.

<https://www.graphpad.com/features>

**Discussion:**
**[R1] = The discussion is also mostly on-point, well done.
However, I believe that the use of elastic band to assess dynamic force is VERY interesting. The authors should elaborate on this potential practical application of the device, and point out that other similar devices have NOT been validated in this way (typically only isometric outputs).**

[A] = Thank you for the acknowledgment and the recommendation. We have added a section to the manuscript on practical applications, highlighting this point.

Finally, the authors would like to thank all the reviewer's contributions that have helped

to improve this article.

**Responses to Reviewer #2**

Please note that:

**[R2] = comments of Reviewer #2.**

[A] = answers of the authors.

**[R2] =** The research is very interesting and useful for all people using this type of devices. According to the Reviewer, in future similar studies, the number of the study group should be increased. A control group consisting of, for example, non-exercising people could also be included. To check whether these measurements are equally accurate in both groups.

[A] = Thank you for your input. We are aware that the sample size was small, and we could add this as a limitation in the manuscript. However, we have calculated the statistical power of our results for correlation analysis with this sample. Using the lowest correlation seen in the results (0.94, Fpeak at 120BPM) for the sample size used, the resultant statistical power is 0.98. This is higher than the usual 0.8 recommended power.

The power calculations were performed using the R open-source software through the 'pwr' library, as indicated in the following code snippet. library(pwr): pwr.r.test Power calculations for correlation test

pwr.r.test(n = 13, r = 0.94, sig.level = 0.001, power = NULL)

n = 13

r = 0.94

sig.level = 0.001

***power = 0.98372***

Finally, the authors would like to thank all the reviewer's contributions that have helped to improve this article.
